# Waardenburg Syndrome Expression and Penetrance

**Published:** 2017-12-10

**Authors:** Myeshia V. Shelby

**Affiliations:** Department of Genetics and Human Genetics, Howard University Graduate School, Howard University, USA

**Keywords:** Nonsense-Mediated Decay, Variable Expressivity, Reduced Penetrance, Nonsense Mutation, Alternative Splicing, Waardenburg Syndrome

## Abstract

Through a combination of in silico research and reviews of previous work, mechanisms by which nonsense-mediated mRNA decay (NMD) affects the inheritance and expressivity of Waardenburg syndrome is realized. While expressivity and inheritance both relate to biochemical processes underlying a gene’s function, this research explores how alternative splicing and premature termination codons (PTC’s) within mRNAs mutated in the disease are either translated into deleterious proteins or decayed to minimize expression of altered proteins. Elucidation of splice variants coupled with NMD perpetuating the various symptoms and inheritance patterns of this disease represent novel findings. By investigating nonsense mutations that lie within and outside the NMD boundary of these transcripts we can evaluate the effects of protein truncation versus minimized protein expression on the variable expressivity found between Type I and Type III Waardenburg syndrome, *PAX3,* while comparatively evaluating *EDN3* and *SOX10’s* role in inheritance of Type IV subtypes of the disease. This review will demonstrate how alternative splicing perpetuates or limits NMD activity by way of PTC positioning, thereby affecting the presentation of Waardenburg syndrome.

## Introduction

Waardenburg syndrome (WS) is typically inherited as an autosomal dominant disease that affects an estimated 1 in 40,000 people but has also been seen in recessive cases^1^. The National Organization for Rare Disorders^2^ states that the disease accounts for 2 to 5 percent of all cases of congenital hearing loss and has a spectrum of severity ranging from pigmentation anomalies to limb deformities and neurological impairment (Table 1). This broad range of expression in symptoms is a result of the different mutations involved in creating the diseased state per The U.S. National Library of Medicine^3^.

**Figure 1.**
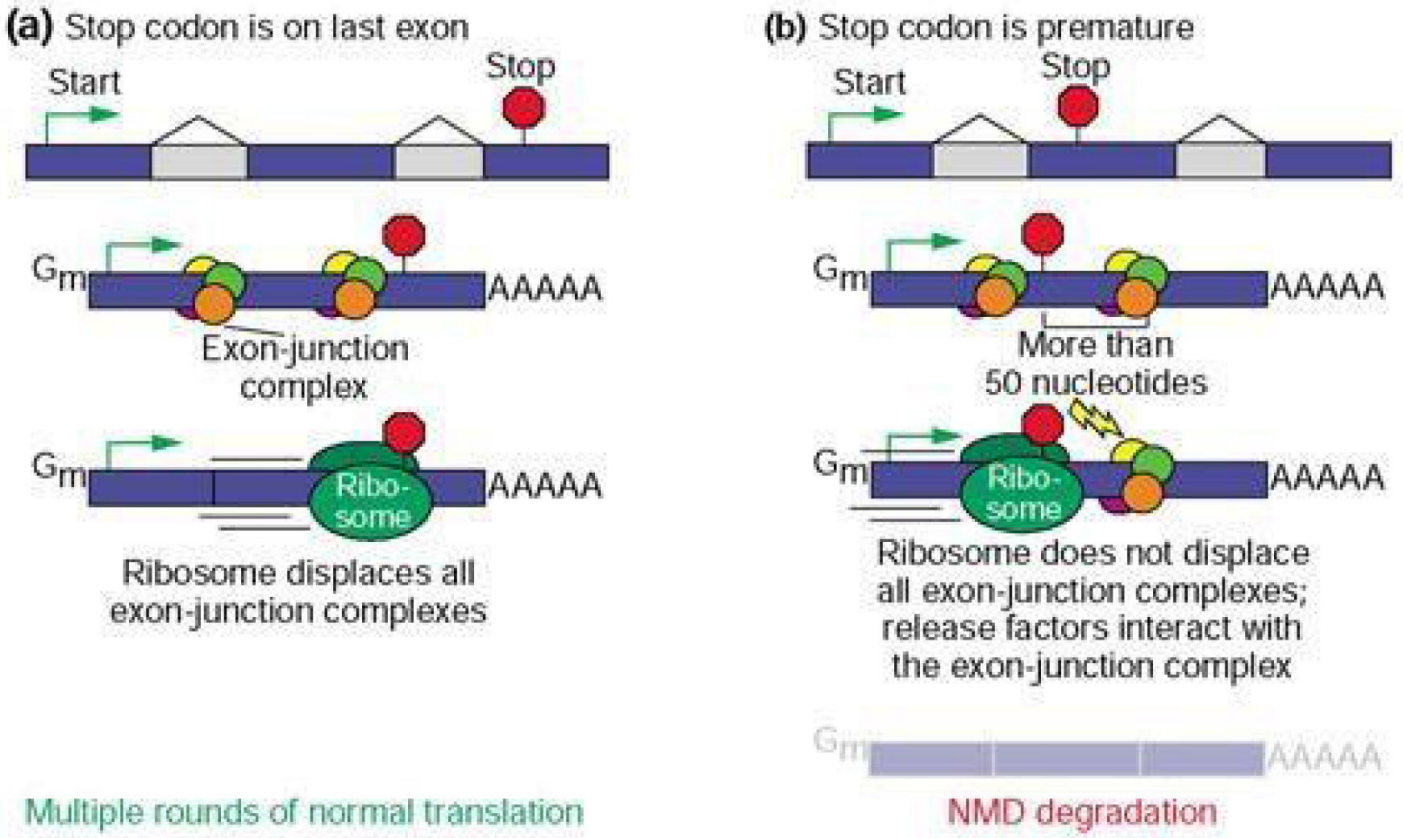
Mechanisms of NMD Surveillance^9^ Termination codons that appear in the last exon (a) result in multiple rounds of normal translation due to the removal of all exon-junction complexes by the ribosome. Termination codons that appear prematurely (b), upstream of the last exon, generally trigger NMD degradation due to the presence of exon-junction complexes downstream of the PTC that are not displaced by ribosomes and interact with release factors.

Nonsense mediated decay (NMD) is a surveillance mechanism incorporated into the transcription/translation pathway of all eukaryotic cells^4^ (Figure 1). This quality control system reduces the accumulation of mRNA containing nonsense mutations and the eventual aberrant polypeptides they may encode^5^. Depending on the function of the resulting protein, NMD could turn out to have downstream phenotypic effects that are either beneficial or harmful to the organism. Advantages of NMD activity occur when the potential protein has more deleterious capabilities than a partially functioning protein, but is prevented from making a phenotypic impact when its mRNA is decayed^6^. Otherwise, NMD causes more overall harm when a partially functioning protein’s mRNA is targeted for degradation and prevents the protein from performing a residual role within the cell^7^. Thus, NMD can impact the expression and penetrance of human genetic diseases depending upon the position of premature termination codons (PTC’s) that potentially limit protein synthesis^8^.

The probability that NMD will alter phenotype and/or contribute to the heritability of WS depends upon: (1) the molecular lesion within the gene, (2) the location of a nonsense mutant codon within the gene coding region and (3) the elimination of the function of a protein in a disease-related pathway. Mutant stop codons upstream of the “NMD boundary” (50 to 55 bases 5’ of the last exon-exon junction) generally trigger degradation of the affected transcript and limit truncated protein production^9^. The NMD boundary is exemplified by exon-junction complexes (EJC) that remain attached to mRNA in the presence of upstream PTCs, which allow release factors to remove ribosomes and recruit NMD key components^10^. In contrast, mutant stop codons 3’ of this boundary generally make detection by the NMD machinery less efficient, leading to aberrant mRNA translation^11^. Alternative splicing could alter the outcome of suspected NMD targeted transcripts in many ways. Namely, by the repositioning of PTC locations and adjusting exon numbers, protein synthesis can either be enhanced or jeopardized.

Most nonsense mutations that prematurely terminate translation produce truncated proteins that can interfere with the overall function of the gene^8^. This is a key function of NMD surveillance: restricting the accumulation of harmful truncated proteins and their phenotypic effects. Individuals suffering from WS due to nonsense mutations present with variable expressivity^12^ as well as reduced penetrance^13^ that could be ascribed to NMD activity. Accordingly, Inoue et al.^8^ attributed milder phenotypes to nonsense mutations located within internal exons of mRNAs being comparatively unstable to mRNAs with PTCs in their last exons. The Human Gene Mutation Database reveals that 12% of genetic diseases are caused by a single-point mutation that produce PTCs^14^. This does not account for the frameshift or splice site mutations that may also introduce PTCs that are responsible for as many as one-third of cancers and other inherited disorders^15^. By exploring the positions of nonsense mutations within the transcript variants of WS, an understanding of how NMD and splicing variation affects penetrance and expressivity can be reached.

## Background

Waardenburg syndrome presents within the population in four main types that are categorized by either their phenotypic or genotypic characteristics. Most often, the syndrome displays an autosomal dominant pattern of inheritance. However, Read and Newton^16^ illustrated that Type II WS and Type IV WS (WS2 and WS4) can exhibit autosomal recessive inheritance in those affected. Type I WS and Type III WS (WS1 and WS3) are caused by *PAX3* gene mutations on chromosome 2q36.1, which is responsible for encoding the Paired box 3 transcription factor^17^ that is involved in neural crest cell border induction at the neural plate. Individuals with mild pigmentation loss, dystopia canthorum (broad nasal ridge) and synophrys (unibrows) are categorized with WS1, while those who have hearing loss, hand and arm disfigurement, and pigmentation anomalies are considered to be suffering from WS3 (Klein-Waardenburg syndrome). Mutations in the *Microphthalmia-associated Transcription Factor (MITF)* gene, mapped to chromosome 3p13, result in Type II WS (WS2), which is similar to WS1 apart from dystopia canthorum, and those with WS2 are more often deaf but do not present with abnormally small eyes as seen in the microphthalmia disorder itself which is often times not inherited and do not involve the genes contributing to WS^18^. Sanchez-Martin et al.^19^ attributed these differences to the involvement of several alleles along with at least one other locus in manifesting as WS2. Later observations of *SNAIL homolog 2 (SNAI2)* gene mutations on chromosome 8q11, were documented to be more often associated with hearing loss in WS2^20^. Type IV or Waardenburg-Shah syndrome is associated with mutations in either *Sry BOXIO transcription factor (SOX10),* at 22q13.1, *Endothelin 3 (EDN3),* at 20q13, or *Endothelin Receptor Type B (EDNRB)* on chromosome 13q22.3^21,22^. The genes involved in this subtype are all significant in the production of melanocytes as well as enteric ganglia, making WS4 subjects more prone than other subtypes of the disease to amassing symptoms associated with pigmentation anomalies, hearing loss and intestinal problems that coincide with Hirschsprung’s disease (HSCR)^23^.

Neural crest cell differentiation, much like melanocytes, depends upon the instruction of SOX10 to mediate gene expression of bona fide neural crest cells and their resultant regulation. These gene specifications allow for epithelial-mesenchymal transition that yields these cells as a highly migratory, multipotent progenitor population^24^. Beginning during embryogenesis, melanocyte development stems from neural crest cells and becomes specialized for pigment production throughout the body. Transcription factors MITF, PAX3, SNAI2, and SOX10 have been described as early genetic markers for melanocyte differentiation that create and sustain molecular pathways for embryonic melanoblast precursors^25^. However, when mutations occur in any of the genes encoding these proteins there are downstream effects that become apparent in congenital pigmentation disorders such as WS^26^.

There are over 50 autosomal dominant disease-associated mutations in the *PAX3* gene, all of which have been found to disrupt almost every domain of the protein in WS1 and WS3, inhibiting DNA binding of the encoded transcription factors and expression of other genes^27^. Frameshift, nonsense, splice site, point mutations, and even cases of entire gene deletions have all been recorded in this gene. These mutations all lead to similar phenotypes caused by haploinsufficiency and a loss of protein function, which manifests as WS1^28^. Melanocyte survival and limb bud formation are both relatively insensitive to *PAX3* levels owing to the variation in phenotypic expression in those affected. However, in a homozygous state there is much more pigment and hearing loss along with upper musculoskeletal deformities that are mainly associated with Klein-Waardenburg syndrome, WS3^29^. The major difference in phenotypic expression between WS1 and WS3 is the prevalence of typical WS1 characteristics with upper extremity malformations in those with WS3^16^. Also, Barbera et al.^28^ provided that WS3 mutations are usually *de novo* or stem from families of WS1 that are heterozygous for PAX3 deletion mutations.

Unlike WS1 and WS3, WS2 exhibits locus as well as allelic heterogeneity. In addition to the inappropriate expression of *SNAI2* in WS2, Asher and Friedman^30^ presented many alleles of *MITF* in this disorder, which occur as both dominant and recessive mutations. Both genes associated with WS2 are responsible for embryonic neural crest cell migration and melanocyte differentiation. So unsurprisingly, auditory-pigmentation disorders have long been associated with these genes^31^. Neural crest are multipotent, migratory cells that are induced at early vertebrate embryogenesis by molecular signals (i.e. bone morphogenetic protein) from surrounding tissues to activate neural plate border genes (i.e. Pax3). Activation of these genes distinguishes non-neural ectoderm cells from those destined to become the neural tube at neurulation. After neural plate border initiation, transcription of neural crest specifier genes (i.e. SNAI2 and SOX10) are activated by neural plate specifier genes and signaling molecules that allows these cells to undergo epithelial to mesenchymal transition and migrate eventually becoming smooth muscle, craniofacial cartilage and bone, peripheral and enteric neurons and glia as well as melanocytes. MITF protein is part of the basic helix-loop-helix leucine zipper (b-HLH-Zip) family of transcription factors that gets activated in early melanogenesis. Hou and Pavan^32^ reported that basic region mutations resulted in dominant negative effects, and mice heterozygous for this type of mutation developed white spotting or diluted coat color, while mice homozygous for the recessive allele had dimerization defects and developed an entirely white coat. Incidentally, the *MITF* gene has been shown to be regulated by *SOX10* and *PAX3*^31^ and mutations in either one of these genes prohibit expression of *MITF* and subsequent neural crest derived melanocytes. When evaluating *SNAI2* involvement in WS2 as a zinc-finger transcription factor, Julie Schultz^33^ reported that *MITF* trans activates the *SNAI2* promoter and that homozygous deletion mutations must occur at the *SNAI2* loci preventing protein production that manifests the phenotypes seen in WS2. In addition, *SNAI2* activation is reliant upon cooperation from signal molecule pathways and *PAX3* while expression maintenance is dependent upon transcription factors such as SOX10.

Investigations of megacolon mice lead to the discovery of the three neural crest genes responsible for Waardenburg-Shah syndrome, WS4. Matsushima et al.^34^ revealed a novel *EDNRB* gene deletion mutation that inhibited endothelin 3, EDN3, from interacting with its receptor and thus the differentiation of melanocytes and Auerbach’s plexus cells. They also found the phenotypic outcomes of this mutation to be similar to WS4 and transmitted recessively. However, there is an intimate relationship between HSCR and WS4 because of the shared genes between the two disorders, most notably *EDN3* and *EDNRB*. These genes exhibit dosage sensitivity in that homozygous substitution, deletion or nonsense mutations lead to symptoms of both Waardenburg and Hirschsprung’s while heterozygotes show minimal effects or have isolated HSCR^16^. There are over 30 identified dominant gene mutation of *SOX10* that cause WS4, with nonsense and missense being the most abundant mutation type. Bondurand et al.^31^ demonstrated that dominant megacolon mice for *SOX10* mutations interfered with *MITF* expression allowing for these mutations to correlate with type II syndromes as well. SOX10 transcription factors are responsible for prompting the migration of melanocytes and enteric nerves during embryonic development owing to the loss of pigmentation associated with WS and the intestinal blockage of HSCR^35^.

Longer genes are directly correlated to increased transcript variants and are more likely to acquire new mutations^36^. These new mutations possibly manifest as a result of cell division when RNA polymerase and DNA polymerase collide during replication creating fragile sites within the DNA. The likelihood of these types of collisions is minimized in genes with less than 20 kilobases^37^. However, some genes have natural fragile sites of instability such as those with tandem nucleotide repeats, which are also more prone to mutations due to slipped-strand misalignment^38^. Recombination hotspots present another means by which spontaneous mutations could occur within the genome. DNA motifs of some genes are capable of summoning regulatory enzymes that initiate breakpoints for cross-over events to ensue^39^. The odds for DNA misalignment and recombination errors that lead to disease are increased when these motifs are palindromic and/or repetitive sequences^40^. Additionally, Supek and Lehner^41^ demonstrated that DNA repair machinery is less efficient in genes that are not biologically active within a cell. In focusing its action on expressed genes, DNA repair is limited in silenced genes which allows these genes to also accumulate mutations.

## Research question

Will analysis of PTC positions in WS alleles reveal that the NMD pathway accounts for the different phenotypes and clinical presentations that may be further researched toward development of therapeutics?

## Hypothesis

NMD plays a role in the penetrance and expressivity of the distinct types of WS.

## Specific aims

Individuals having Waardenburg syndrome suffer from a wide array of physical characteristics, some of which may be affected by NMD. Even more so, these characteristics represent a wider range of gene mutations. Our aim is to:

1. Identify alleles within WS which may be candidates for NMD.
2. Compare NMD candidate alleles with other WS alleles for their unique functional properties in relation to expressivity and penetrance.
3. Determine the role alternative splicing has in PTC location and NMD potentiation regarding disease presentation.

## Significance

By exploring the possibility that NMD and alternative splicing play a significant role in the penetrance and expressivity found in WS, treatment administration could be revolutionized. Also, the presence of NMD components could be extrapolated upon the manipulation of treatment and/or prevention of other disorders if the introduction or eradication of PTCs could be more beneficial to the overall genome.

## Methodology

First, transcripts of *SOX10, EDN3* and *PAX3* that are responsible for wild-type development were identified and compared to any symptomatic alleles and splice variants connected to the disease. All isoforms were found through the National Center for Biotechnology Information^42^, Ensembl^43^, Uniprot^44^, and GeneCards^45^ websites.

**Figure 2.**
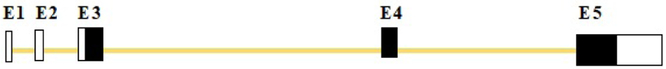
*SOX10* Transcript The 5 exons of SOX10 are denoted by the shaded translated regions and unshaded untranslated regions. Intronic regions are represented in yellow. The translated regions are confined to 3 exons that encode a 466-amino acid protein. Potential PTCs would also be found within these 3 exons.

**Figure 3.**
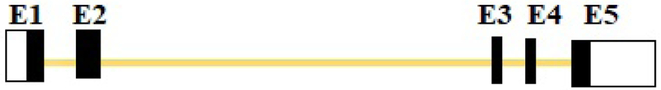
*EDN3* Transcript The 5 exons of EDN3 are denoted by the shaded translated regions and unshaded untranslated regions. Intronic regions are represented in yellow. Translated regions are located within each of the exons and encode a 238-amino acid protein. Potential PTCs would also be found within these exons.

**Figure 4.**
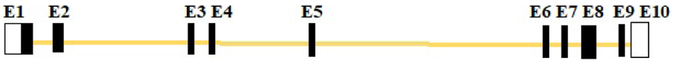
PAX3 Transcript The 10 exons of PAX3 are denoted by the shaded translated regions and unshaded untranslated regions. Intronic regions are represented in yellow. Translated regions are confined to 9 exons and encode a 479-amino acid protein. Potential PTCs would also be found within these exons.

Next, we found human *SOX10* consists of 5 exons (Figure 2) with only 3 exons translated to a 466-amino acid protein^46^. Alternative splicing of the gene allows for four protein encoding isoforms SOX10–001 through SOX10–004. The *EDN3* gene has 5 exons (Figure 3) that encode 238 amino acids, with five possible isoforms: EDN3–001 - EDN3–004 and EDN3 −20 1^23^. There are 10 exons that make up the *PAX3* gene (Figure 4) and eight identified protein isoforms^28^. The genome browsers explicitly provide nucleotide information exon by exon for each isoform as well as disease causing mutations, allowing us to specifically evaluate isoforms manifesting WS phenotypes only.

Each transcript variant was then scrutinized for PTCs in the NMD zone of activation as opposed to other nonsense mutations. Lastly, Journals of Medical Genetics, World Journal of Gastroenterology, American Journal of Human Genetics, Chinese Medical Journal and peer reviewed articles such as: Human Mutation, Human Genetics, Human Molecular Genetics, Seminars in Hearing and Scientific Reports were cross referenced for the associated mutations’ incidence and presentation.

## Results

By reviewing available literature and genome databases, transcripts of WS alleles and the role nonsense mediated decay plays in the disease was revealed. Due to *SOX10* regulation of *MITF* and its involvement in the expressivity of WS4A and WS4C, its transcripts’ nonsense mutations were focused on (Table 2). All SOX10 transcripts investigated contain an NMD candidate mutation that encodes a PTC in place of tyrosine at position 83. This mutation causes WS4A with chronic bowel problems due to the reduction of enteric ganglia found via rectal biopsy and haploinsufficiency of the protein^46^. The SOX10–001 and SOX10–002 transcripts contain mutations that encode a PTC at position 189 instead of the wild type glutamine residue within the targeted NMD boundary. Although these two transcripts have different numbers of exons, the PTCs are found within the NMD boundary in both, essentially making the transcripts equivalent to one another. This particular mutation has also been associated with the less severe WS4A due to haploinsufficiency of the protein^8^. This is consistent with NMD activity in that the nonsense mutations found in the last exon of these transcripts have been reported in those with the more severe phenotype of WS4C and syndromes associated with the complex neurocristopathy disease PCWH^47^. Functional analysis by Southard-Smith et al.^48^ demonstrated that truncation of SOX10 in this 3’ region maintains their high mobility group (HMG] box but eliminates the transactivation domain and hinders proliferation of neural crest and melanocyte transcription factor regulated cells creating a dominant-negative effect when these transcripts escape NMD. Inoue et al.^8^ demonstrated that this effect correlated with DNA binding affinity and the length of truncated proteins; the shorter the protein the more it bound to its target DNA and enhanced the deleterious effect. Furthermore, Inoue et al.^8^ conducted assays with comparative intron-containing minigene constructs and found that NMD takes place whenever an intron is preceded by a PTC and therefore diminishes the dominant-negative presence of a truncated protein *in vivo*. This may be opposed to individuals suffering from WS4C that is described by Pingault et al.^46^ in which individuals with the previously mentioned nonsense mutation that encodes a PTC in place of glutamine at position 189 present with short segment Hirschsprung disease. Pingault et al.^46^ attributed this phenotype to truncated proteins that maintain their (HMG) box, which could manifest within the SOX10–003 transcript because the PTC is in the last exon and possibly escapes NMD as opposed to the same mutation in SOX10–001 and SOX10–002 that produces reduced mRNA levels^8^. This nonsense mutation is not found in the SOX10–004 isoform that consists of 179 residues. This team proposed that the dose-dependent method by which SOX10 mutations inhibit cell activation and proliferation is complicated by NMD activity and the degradation of the dominant-negative mRNA, which creates the haplo-insufficient state. Overall, only the SOX10–002 transcript presented a 3:4 ratio of PTCs within the NMD boundary to those outside the boundary while the other transcripts were 1:1 in this respect. Each nonsense mutation in these transcripts was fully penetrant for the diseased state, though expression variation was due to the location of nonsense mutations outside of the NMD boundary that allows truncated proteins to be synthesized at lengths corresponding to the PTC. Together, the data are consistent with gradations of dominant-negative effects that coincide with the various extreme phenotypes of WS4.

The EDN3 ligand was investigated for its role in WS inheritance of types 4A and 4B (Table 3). All isoforms of this gene have nonsense mutations within the NMD boundary in exon 3 that encodes a PTC in place of cysteine at position 169 that could induce NMD and no PTCs outside of the boundary. These mutations are associated with WS4A and a heterozygous transversion of cytosine to adenine^21^. Evaluations of this gene by Pingault et al.^21^ postulated that these nonsense mutations could interfere with the enzymatic cleaving of preproendothelin to proendothelin, yielding an inactive protein in the absence of NMD. However, alternative splicing of *EDN3* creates only 4 exons of the transcript EDN3–004 which contains a PTC at position 169 in exon 3, but a shorter 3’ untranslated region (UTR) of 406 nucleotides; whereas, all other EDN3 transcripts studied contain much larger 3’ UTRs of between 1290 and 1660 nucleotides. Yepiskoposyan et al.^49^ identified mechanisms that make transcripts with longer 3’ UTRs more likely to increase NMD efficiency. Whenever ribosomes stall at PTCs that are at a greater distance from the poly(A) binding protein (PABP), which is responsible for managing the elements involved in regular termination, it allows time for the NMD key component UPF1 to bind instead and increase NMD potential. More so, Singh et al.^50^ demonstrated that longer endogenous UTRs could antagonize NMD by preventing release factors from interacting with their target proteins and the disassociation of ribosomes from mRNA allowing translation to ensue. Later work performed by Hogg and Goff^51^ demonstrated that longer 3’ UTRs downstream of PTCs are necessary for recognition by UPF1 which is the core of NMD machinery. This concept makes degradation by NMD more likely to occur in all but one of the EDN3 transcripts and the consequent decreased expression of symptomatic protein may account for the incomplete penetrance documented by Pingault et al.^21^. Otherwise, patients may present with the haplo-insufficiency seen in WS4A that results from a truncated protein which deletes the biologically active domain as described by Svensson et al.^52^. Furthermore, Dupin et al.^53^ documented a dose-dependent relationship between EDN3 transcripts and the stimulation and proliferation of melanocytes and neural crest cells. This may explain the reduced penetrance of WS4A as well as the recessive inheritance of WS4B when we account for the alternative splicing of the gene that could elicit different amounts of mRNA.

Transcripts of *PAX3* were determined as the primary causes of WS1 and WS3^27^. All identified transcripts contained a nonsense mutation within the NMD boundary in exon 3 encoding a PTC in place of lysine at position 139 that could undergo decay (Table 4) and no other PTCs. These variants all demonstrated full penetrance of WS1, which is less severe than the phenotypes that define WS3. However, Yang et al.^54^ presented two individuals suffering from WS1 with nonsense mutations in exon 5, one with a PTC in place of serine at position 209 and the other with a PTC in place of arginine at position 223. Both patients presented with characteristic features of WS1 and *PAX3* mutations that could be targeted by NMD, but based on the overall structure of the protein were predicted to encode truncated proteins that eliminate the homeodomain thus preventing DNA binding and targeted gene expression^55^. The translation of truncated proteins in these individuals may indicate a decrease in NMD efficiency that is suggestive of codon bias and increased mRNA stability. Highly translated gene locations can be predicted by the increased presence of ribosomes^56^, while work performed by Presnyak et al.^57^ demonstrated that the destabilization of decay mechanisms could be attributed to the close association of codon optimality and the translocation of ribosomes that allow translation to occur. In other findings, Xiao-Li et al.^58^ conducted western blot analysis of mouse models containing nonsense mutations encoding a PTC instead of lysine at position 107. This mutation occurs in the 3’ distal end of exon 2 and was shown to contain no truncated or normal protein properties in the homozygous state, which is indicative of loss of function. The absence of heterozygote extreme phenotypic severity that would be seen in homozygous mutations of truncated PAX3 demonstrates that the gene does not have dominant-negative properties^59^. This is supported by findings of Hol et al.^60^ that a nonsense mutation inserted into exon 6 that replaces glutamine for a PTC at position 282 encodes a truncated protein that includes all conserved domains. Interestingly, patients with this mutation showed similar clinical traits as those with mutations affecting all or part of the conserved domains, emphasizing the importance of the C-terminus for wild-type development and the variation in gene expression due to the type of mutation and the subsequent protein dosage of tissue specific transcripts.

## Discussion

Variable expressivity and reduced penetrance are genetic terms that define biological and clinical phenotypes of a disease. An individual with the diseased state genotype may not necessarily present with the disease trait characteristics when penetrance is involved, especially when the genes in question encode dosage-sensitive proteins. This could be due to an additional nonsense mutation that causes decay of the affected gene transcript that would otherwise increase phenotypic penetrance if protein translation ensued. Alternatively, variable expressivity describes instances when all affected individuals encounter different degrees of phenotypic expression that could be due to decay, microRNA availability, alternative splicing and/or amino-acylated tRNA. When different transcripts are expressed in different tissues they may present unique decay properties that allow various levels of expression in those affected. Here we asked whether NMD plays a role in the molecular pathogenesis of expressivity and inheritance of WS and how alternative splicing impacts mRNA degradation. It is our understanding that the heterogenous nature of the genes involved undergo various types of mutations, of which a subset could represent NMD precursors. In analyzing these alleles through genomic databases, we were able to improve upon our understanding of the pathogenic pathway by - focusing on the pleiotropic genes of WS, identifying transcripts that may be degraded by NMD, and associating the diseased state genotype with phenotypic outcome by analyzing available data corresponding to molecular activity.

Previous work has shown that *PAX3* mutations are involved in both WS1 and WS3^61^; however, those suffering with much more drastic phenotypes are categorized in the latter subtype^62^. We observed that all nonsense mutations of PAX3 transcripts lead to phenotypes consistent with WS1 only, which leads us to believe that these individuals suffer from a haploinsufficiency as opposed to the total loss of protein function in the mutated homolog as seen in WS3. The haploinsufficiency and corresponding phenotype of WS1 could be derived from the fully functioning protein of the wild type homolog and the partial function of the truncated protein that is synthesized in affected heterozygotes. However, WS3 may manifest in individuals harboring mutated PAX3 transcripts that undergo decay and must rely on the wild type homolog protein only. All isoforms investigated showed candidacy for NMD and the subsequent limitation of protein synthesis that is associated with both WS1 and WS3^22^. Case studies by Yang et al.^54^ provided the prospect that some NMD eligible nonsense mutation transcripts do not undergo decay but encode proteins that contribute to the WS1 phenotype. We know this is possible because, PAX3 relies on its highly-conserved domains to regulate other genes as a transcription factor. Any disruption of these domains, especially the C-terminus, yields a loss of gene function that is present in these Waardenburg syndrome subtypes. Cases of WS3 have been documented to result from compound heterozygous and homozygous nonsense mutations which is consistent with our findings of PAX3 function^63^.

The various inheritance patterns of this disease presented more concerns. The biochemical processes which underline the modes of inheritance involve protein synthesis function and availability. Because proteins can exhibit either dose-dependent loss of function or dominant-negative inhibition, protein quality and quantity contribute to the way in which diseases are acquired^64^. A heterozygous carrier of an NMD type mutated gene can still rely on the wild-type allele for proper function without interference from dominant-negative effects of the mutated protein. This could result in the autosomal recessive pattern of inheritance seen in WS4B. This feature of the disease led us to assess *EDN3* alleles and identify possible candidates for this inheritance pattern as opposed to those that allow for maladaptive protein expression. Edery et al.^65^ identified individuals with nonsense mutations within the NMD boundary, but functional in vitro assays by Pingault et al.^21^ illustrated the presence of non-functional proendothelin protein. Although more research is needed, we speculated that only 1/5^th^ of the transcripts were likely penetrant to WS4A due to limited NMD activity. By relying on experiments conducted by Hogg and Goff^51^, we believe that the extensive length of the 3’ UTRs of the other transcripts containing PTCs allow them to summon UPF1 quicker than PABP is able to bind and efficiently terminate translation. This possibility explains why some *EDN3* mutations are also inherited recessively and demonstrate variable expressivity when referring to Dupin et al.’s^53^ work on the gene’s dosage efficacy on cell proliferation.

Alleles of *SOX10* were also examined as a hierarchal pathway gene and for its role in accentuating the symptoms found between the subtypes of WS4. *SOX10* is responsible for activating *MITF* and regulating the ensuing neural crest mobility cascade^66^. In the absence of NMD in these transcripts, a dominant negative response ensues that causes the truncated protein to bind DNA with an affinity that inversely correlates to its length^8^. This binding interferes with wild-type transcription, in heterozygotes, and is allowed to wreak havoc on migrating neural crest cells in type 4C of WS and implications in Type 2 (WS2). Alternatively, if PTCs occur within the NMD boundary and are targeted for decay, a less severe phenotype could ensue due to avoidance of the negative consequence of a truncated protein.

## Conclusion

The explication of NMD provides information on the biochemical pathway and the molecular etiology of complex monogenic inheritance patterns and therefore identifies mechanisms of reduced penetrance and variable expressivity. This thorough review of transcripts associated with the patterns encompassing WS has presented refreshed concepts in which alternative splicing alters the location of PTCs in relation to the last exon making degradation dependent upon these splice variations^67^. Nonsense mutations that occur early in a gene’s open reading frame could decrease expression of dominant-negative genes and to the same degree minimize penetrance of dosage-sensitive genes.

We identified methods by which NMD could function as a phenotypic modulator in SOX10 transcripts between subtypes 4A and 4C of WS. Those inheriting PTCs beyond the decay boundary presented with 4C and the coinciding nerve anomalies. Bondurand et al.^68^ suggests this is because of the dominant negative power of the synthesized protein as opposed to the haploinsufficiency found in 4A transcripts with PTCs within the decay boundary. This contrast in expression is perpetuated during the migration of embryonic nerve cells that populate the colon. Nonsense mutation SOX10 transcripts that do not undergo NMD interfere with wild-type transcriptional activity in a quantifiable manner, so when these transcripts are targeted for decay the wild-type allele is able to mitigate the impacts of transcription interference that would otherwise ensue. This gene appears most probable of all investigated to be impacted by alternative splicing in relation to decay. Our findings indicated that the expressivity associated with SOX10 in WS can be attributed to both NMD and alternative splicing.

Penetrance of WS4 is influenced by the melanocyte differentiation ligand EDN3. The gene encoding this protein exhibits dosage sensitivity and NMD activity may be augmented by the length of 3’ UTRs of these transcripts^69^. A phenomenon such as this could contribute to recessive inheritance and explain why heterozygous carriers of this mutation present as either non-symptomatic or with WS4A. Also, some *EDN3* transcripts could be spliced so that NMD detection is increased and the presence of a wild type ligand in these individuals would elicit enough melanocyte activity for them to be unaffected.

In attempt to expand our knowledge of the role NMD plays in symptom acquisition and combat congenital hearing loss through gene therapy, we have speculated means by which the location of PTCs could exacerbate disease expression and penetrance. We have also shown the futility of NMD in the PAX3 gene as it relates to these disease components. The biochemical properties of disease discussed here allow an array of potential targets to be acted upon in hopes of minimizing and/or preventing symptomatic outcomes. With the genetic manipulation techniques we now have at our disposal, these and other splice variants could be further investigated in their relation to NMD and disease. By utilizing developments such as CRISPR-Cas9 genome editing we could essentially introduce PTCs or splice sites that make NMD more likely to occur if it is deemed more beneficial for the long-term livelihood of an organism. In addition, mechanisms such as mRNA trans-splicing could remove PTCs that are reportedly symptomatic and replace them with corrective sequences.

## Figures and Tables

**Table 1:** Diseased State and Clinical Presentations of Waardenburg Syndrome.

CATEGORY	LOCI	GENE(S) INVOLVED	INHERITANCE PATTERN	PHENOTYPE
Type 1	2q36.1	PAX3	Autosomal Dominant	Broad nasal root, Unibrow, Mild pigment anomalies
Type 2	3ql3 8qll	*MITF* *SNAI2*	Autosomal Dominant, Autosomal Recessive	Deafness, Unibrow, Mild pigment anomalies, Het-erochromia
Type 3 Klein- Waardenburg	2q36.1	PAX3	Autosomal Dominant	Broad nasal root, Hearing loss, Hand and Arm disfigurement, Pigment anomalies
Type 4A Waardenburg-Shah	22ql3.1 20ql3 13q22.3	*SOX10* *EDN3* *EDNRB*	Autosomal Dominant	Pigmentation anomalies, hearing loss, constipation
Type 4B Waardenburg-Shah	20ql3 13q22.3	*EDN3* *EDNRB*	Autosomal Recessive	Pigmentation anomalies, hearing loss, Hirsch sprung
Type 4C Waarde burg-Hirschsprung	22ql3.1	SOX10	Autosomal Dominant	Pigmentation anomalies, hearing loss, Hirschsprung - related severe constipation, neurological impairments

**Table 2:** Human SOX10 Transcript Descriptions.

TRANSCRIPT	EXON AMOUNT	AMINO ACID	PTC LOCATION
SOX10	5	466	Wild Type
SOX10–001 398 variations	5	466	3 - Tyr83X,Trpl42X 4 - Glul89X,Tyr207X 5 - Gln250X, Ser251X, Ser376X, Gln377X
SOX10–002 398 variations	4	466	2 - Tyr83X 3 - Glul89X,Tyr207X 4 - Gln250X Ser251X, Gln377X, Ser379X
SOX10–003 170 variations	3	213	2 - Tyr83X,Trpl42X 3 - Glul89X,Tyr207X
SOX10–004 169 variations	4	179	2 - Glu66X,Tyr84X 3 - Glnl27X, Serl28X

**Table 3:** Human EDN3 Transcript Descriptions.

TRANSCRIPT	EXONS	AMINO ACIDS	PTC LOCATION
EDN3	5	238	WildType
EDN3–001 406 variations	5	238	3 - Cysl69X
EDN3–201 406 variations	6	238	3 - Cysl69X
EDN3–002 367 variations	5	219	3 - Cysl69X
EDN3–003 387 variations	4	224	3 - Cysl69X
EDN3–004 341 variations	4	192	3 - Cysl69X

**Table 4:** Human PAX3 Transcript Descriptions.

TRANSCRIPT	EXON AMOUNT	AMINO ACIDS	PTC LOCATION
PAX3	10	479	Wild Type
PAX3–001 596 variations	8	403	3 - Lysl39X and 5 - Arg223X
PAX3–002 597 variations	9	407	3 - Lysl39X and 5 - Arg223X
PAX3–003 358 variations	5	206	3 - Lysl39X
PAX3–004 684 variations	9	484	3 - Lysl39X and 5 - Arg223X
PAX3–005 367 variations	4	215	3 - Lysl39X
PAX3–006 675 variations	8	479	3 - Lysl39X and 5 - Arg223X
PAX3–008 698 variations	10	505	3 - Lysl39X and 5 - Arg223X

## References

[1] Vicente-Dueñas C, Bermejo-Rodríguez C, Pérez-Caro M, Atlas of Genetics and Cytogenetics in Oncology and Haematology. [Internet]. Spain: IBMCC; 2005 [2017]. http://atlasgeneticsoncology.org/Kprones/WaardenburgID10089.html.

[2] Milunsky J National Organization for Rare Disorders. [Internet]. Danbury, CT: NORD; 1987 [2017]. https://rarediseases.org/rare-diseases/waardenburg-syndrome/.

[3] National US Library of Medicine. [Internet]. Bethesda, MD: The Library; 2016 [2017]. https://ghr.nlm.nih.gov/condition/waardenburg-syndrome#genes.

[4] Brogna S, Wen J. Nonsense-mediated mRNA decay (NMD) mechanisms. Nat Struct Mol Biol. 2009; 16(2): 107–13. 19190664 10.1038/nsmb.1550

[5] Hentze M and Kulozik A. A Perfect Message: RNA Surveillance and Nonsense-Mediated Decay. Cell. 1999; 96(3): 307–10. 10025395 10.1016/s0092-8674(00)80542-5

[6] Pulak R, Anderson P. mRNA surveillance by the Caenorhabditis elegans smg genes. Genes & Dev. 1993; 7(10): 1885–97. 8104846 10.1101/gad.7.10.1885

[7] Mühlemann O, Eberle AB, Stalder L, Recognition and elimination of nonsense mRNA. Biochim Biophys Acta. 2008; 1779(9): 538–49. 18657639 10.1016/j.bbagrm.2008.06.012

[8] Inoue K, Khajavi M, 0hyama T, Molecular mechanism for distinct neurological phenotypes conveyed by allelic truncating mutations. Nat Genet. 2004; 36(4): 361–9. 15004559 10.1038/ng1322

[9] Hillman Tyler R, Green, An unappreciated role for RNA surveillance. [Open-Access Photo.] Genome Biology. 2004; 5:R8. 14759258 10.1186/gb-2004-5-2-r8 PMC395752

[10] Metze S, Herzog V, Ruepp M, Comparison of EJC-enhanced and EJC-independent NMD in human cells reveals two partially redundant degradation pathways. RNA. 2013; 19(10): 1432–48. 23962664 10.1261/rna.038893.113 PMC3854533

[11] Hall GW, Thein S. Nonsense codon mutations in the terminal exon of the β-globin gene are not associated with a reduction in β-mRNA accumulation: A mechanism for the phenotype of dominant β-thalassemia. Blood. 1994; 83(8): 2031–7. 8161774

[12] Syrris P, Carter N, Patton M. Novel nonsense mutation of the endothelin-B receptor gene in a family with Waardenburg-Hirschsprung disease. AJMJ. 1999; 87(1): 69–71. 10528251

[13] Inoue M, Okada A, Hosoda K, Mutational analysis of the endothelin-B receptor gene in Japanese Hirschsprung’s disease. J Pediatr Surg. 1998; 33: 1206–08. 9721987 10.1016/s0022-3468(98)90151-8

[14] Cooper DN, Ball EV, Stenson PD, The Human Gene Mutation Database. [Internet]. Cardiff: HGMD®; 2007 [2017]. http://www.hgmd.cf.ac.uk/ac/index.php.

[15] Kniffin C, McKusick V. Online Mendelian Inheritance in Man. [Internet]. Baltimore, MD: Johns Hopkins University; 1986 [2017]. https://www.omim.org/.

[16] Read A, Newton V. Waardenburg syndrome. J Med Genet. 1997; 34(8): 656–65. 9279758 10.1136/jmg.34.8.656 PMC1051028

[17] Pingault V, Ente D, Dastot-Le Moal F, Review and update of mutations causing Waardenburg syndrome. Hum Mut. 2010; 31(4): 391–406. 20127975 10.1002/humu.21211

[18] Verma A, FitzPatrick D. Anophthalmia and microphthalmia. J Rare Dis. 2007; 2(11): 47–54. 10.1186/1750-1172-2-47 PMC2246098 18039390

[19] Sanchez-Martin M, Rodriquez-Garcia A, Perez-Losada J, *SLUG* (*SNAI2*) deletions in patients with Waardenburg disease. Hum Mol Genet. 2002; 11(25): 3231–6. 12444107 10.1093/hmg/11.25.3231

[20] Song J, Feng Y, Acke FR, Hearing loss in Waardenburg syndrome: a systematic review. Clin Genet. 2016; 89(4): 416–25. 26100139 10.1111/cge.12631

[21] Pingault V, Bondurand N, Lemort N, A heterozygous *endothelin 3* mutation in Waardenburg-Hirschsprung disease: is there a dosage effect of *EDN3*/*EDNRB* gene mutations on neurocristopathy phenotypes. J Med Genet. 2001; 38(3):205–9. 11303518 10.1136/jmg.38.3.205 PMC1734825

[22] Kubic J, Young K, Plummer R, Pigmentation PAX-ways: The role of Pax3 in melanogenesis, melanocyte stem cell maintenance, and disease. Pigment Cell Melanoma Res. 2008; 21(6): 627–45. 18983540 10.1111/j.1755-148X.2008.00514.x PMC2979299

[23] Duan X, Zhang X, Li G. Clinical relationship between EDN-3 gene, EDNRB gene and Hirschsprung’s disease. World J Gastroenterol. 2003; 9(12): 2839–42. 14669347 10.3748/wjg.v9.i12.2839 PMC4612066

[24] Simões-Costa M, Bronner M. Establishing neural crest identity: a gene regulatory recipe. Development. 2015; 142: 242–57. 25564621 10.1242/dev.105445 PMC4302844

[25] Thomas A, Erickson C. The making of a melanocyte: the specification of melanoblasts from the neural crest. Pigment Cell Melanoma Res. 2008; 21(6): 598–610. 19067969 10.1111/j.1755-148X.2008.00506.x

[26] Hornyak T, Hayes D, Chiu L, Transcription factors in melanocyte development: distinct roles for Pax-3 and Mitf. Mech Dev. 2001; 101(1–2): 47–59. 11231058 10.1016/s0925-4773(00)00569-4

[27] Hoth C, Milunsky A, Lipsky N, Mutations in the paired domain of the human PAX3 gene cause Klein-Waardenburg syndrome as well as Waardenburg syndrome type I. Am J Hum Genet. 1993; 52(3): 455–462. 8447316 PMC1682157

[28] Barbera T, Barbera M, Cloutierd T, PAX3 gene structure, alternative splicing and evolution. Gene. 1999; 237(2): 311–19. 10521655 10.1016/s0378-1119(99)00339-x

[29] Bedell M, Largaespada D, Jenkins N, Mouse models of human disease. Part II: Recent progress and future directions. Genes Dev. 1997; 11(1): 11–43. 9000048 10.1101/gad.11.1.11

[30] Asher J, Friedman T. Mouse and hamster mutants as models for Waardenburg syndromes in humans. J Med Genet 1990; 27: 618–626. 2246770 10.1136/jmg.27.10.618 PMC1017240

[31] Bondurand N, Pingault V, Goerich D, Interaction among *SOX10*, *PAX3* and *MITF*, three genes altered in Waardenburg syndrome. Hum Mol Genet. 2000; 9(13): 1907–17. 10942418 10.1093/hmg/9.13.1907

[32] Hou L, Pavan W. Transcriptional and signaling regulation in neural crest stem cell-derived melanocyte development: do all roads lead to Mitf. Cell Res. 2008; 18: 1163–76. 19002157 10.1038/cr.2008.303

[33] Schultz J Waardenburg Syndrome. Semin Hear. 2006; 27(3): 171–81.

[34] Matsushima Y, Shinkai Y, Kobayashi Y, A mouse model of Waardenburg syndrome type 4 with a new spontaneous mutation of the endothelin-B receptor gene. Mamm Genome. 2002; 13(1): 30–5. 11773966 10.1007/s00335-001-3038-2

[35] Pingault V, Girard M, Bondurand N, SOX10 mutations in chronic intestinal pseudo-obstruction suggest a complex physiopathological mechanism. Hum Genet. 2002; 111(2): 198–206. 12189494 10.1007/s00439-002-0765-8

[36] Grishevich V, Yanai I. Gene length and expression level shape genomic novelties. Genome Res. 2014; 24(9): 1497–1503. 25015383 10.1101/gr.169722.113 PMC4158763

[37] Zylka M Length matters: Disease implications for long genes. [Internet]. University of North Carolina: Spectrum; 2013 [2017]. https://spectrumnews.org/opinion/viewpoint/length-matters-disease-implications-for-long-genes/.

[38] Myers P Tandem Repeats and Morphological Variation. Nature Education. 2007; 1(1): 1.

[39] Nassiri I, Azadian E, Masoudi-Nejad A. A Sequence Motif Associated with Intrinsic Mutation Hot-Spots in Human Cancers. J Proteomics Bioinform. 2013; 6: 183–86.

[40] Purandare S, Patel P. Recombination HotSpots and Human Disease. Genome Res. 1997; 7: 773–86. 9267802 10.1101/gr.7.8.773

[41] Supek F, Lehner B. Differential DNA mismatch repair underlies mutation rate variation across the human genome. Nature. 2015; 521(7550): 81–84. 25707793 10.1038/nature14173 PMC4425546

[42] National Center for Biotechnology Information (US). NCBI Gene Database [Internet]. Bethesda (MD): National Center for Biotechnology Information (US); 2015 [2017]. https://www.ncbi.nlm.nih.gov/books/NBK3831.2017.

[43] Yates A, Akanni W, Amode M, Nucleic Acids Residues [Internet]. Cambridge, UK: Ensembl; 2016 [2017]. 44 Database issue: D710–6. http://useast.ensembl.org/info/about/index.html.

[44] The UniProt Consortium. UniProt: the universal protein knowledgebase [Internet]. Cambridge, UK Nucleic Acids Res; 2002 [2017]. 45: D158–D169. https://academic.oup.com/nar/article/45/D1/D158/2605721. 27899622 10.1093/nar/gkw1099 PMC5210571

[45] Lancet D, Safran M, Rosen N, GeneCards [Internet]. Israel The Weizmann Institute of Science; 1996 [2017]. www.genecards.org.

[46] Pingault V, Bondurand N, Kuhlbrodt K, SOX10 mutations in patients with Waardenburg-Hirschsprung disease. Nat Genet. 1998; 18(2): 171–3. 9462749 10.1038/ng0298-171

[47] Touraine R, Attie-Bitach T, ManLOceau E, , Neurological phenotype in Waardenburg syndrome type 4 correlates with novel SOX10 truncating mutations and expression in developing brain. Am J Hum Genet. 2000; 66(5): 1496–1503. 10762540 10.1086/302895 PMC1378013

[48] Southard-Smith E, Angrist M, Ellison J, , The Sox10Dom mouse: modeling the genetic variation of Waardenburg-Shah (WS4) syndrome. Genome Res. 1999; 9: 215–225. 10077527

[49] Yepiskoposyan H, Aeschimann F, Nilsson D, Autoregulation of the Nonsense-Mediated mRNA Decay Pathway in Human Cells. RNA. 2011; 17(12): 2108–18. 22028362 10.1261/rna.030247.111 PMC3222124

[50] Singh G, Rebbapragada I, Lykke-Andersen J. A Competition between Stimulators and Antagonists of Upf Complex Recruitment Governs Human Nonsense-Mediated mRNA Decay. PLoS Biol. 2008; 6(4): e111. 18447585 10.1371/journal.pbio.0060111 PMC2689706

[51] Hogg J, Goff S. Upf1 senses 3’UTR length to potentiate mRNA decay. Cell. 2010; 143(3): 379–389. 21029861 10.1016/j.cell.2010.10.005 PMC2981159

[52] Svensson P, Von Tell D, Molander M, A Heterozygous Frameshift Mutation in the Endothelin-3 (EDN-3) Gene in Isolated Hirschsprung’s Disease. Pediatr Res. 1999; 45(5 Pt 1): 714–7. 10231870 10.1203/00006450-199905010-00018

[53] Dupin E, Glavieux C, Vaigot P, Endothelin 3 induces the reversion of melanocytes to glia through a neural crest-derived glial-melanocytic progenitor. Proc Natl Acad Sci U S A. 2000; 97(14): 7882–7887. 10884419 10.1073/pnas.97.14.7882 PMC16639

[54] Yang S, Cao J, Zhang R, Nonsense mutations in *PAX3* gene cause Waardenburg syndrome type I in 2 Chinese patients. Chin Med J 2007; 120(1): 46–9. 17254487

[55] Tassabehji M, Newton V, Leverton K, PAX3 gene structure and mutations: close analogies between Waardenburg syndrome and the Splotch mouse. Hum Mol Genet. 1994; 3(7): 1069–74. 7981674 10.1093/hmg/3.7.1069

[56] Ingolia N, Ghaemmaghami S, Newman J, Genome-Wide Analysis in Vivo of Translation with Nucleotide Resolution Using Ribosome Profiling. Science. 2009; 324(5924): 218–23. 19213877 10.1126/science.1168978 PMC2746483

[57] Presnyak V, Alhusaini N, Chen Y, Codon optimality is a major determinant of mRNA stability. Cell. 2015; 160(6): 1111–24. 25768907 10.1016/j.cell.2015.02.029 PMC4359748

[58] Xiao-Li G, Hai-Bin R, Yan L, Identification of a novel nonsense mutation on the Pax3 gene in ENU-derived white belly spotting mice and its genetic interaction with c-Kit. Pigment Cell & Melan Res. 2010; 23(2): 252–62. 10.1111/j.1755-148X.2010.00677.x 20095975

[59] Zlotogora J, Lerer I, Bar-David S, Homozygosity for Waardenburg syndrome. Am J Hum Genet. 1995; 56(5): 1173–78. 7726174 PMC1801439

[60] Hol F, Geurds M, Cremers C, Identification of Two PAX3 Mutations Causing Waardenburg Syndrome, One Within the Paired Domain (M62V) and the Other Downstream of the Homeodomain (Q282X). Hum Mutat. 1998;Suppl 1: S145–7. 9452070 10.1002/humu.1380110149

[61] Wollnik B, Tukel T, Uyguner O, Homozygous and heterozygous inheritance of PAX3 mutations causes different types of Waardenburg syndrome. Am J Med Genet A. 2003; 122A(1): 42–5. 12949970 10.1002/ajmg.a.20260

[62] Tekin M, Bodurtha J, Nance W, Waardenburg syndrome type 3 (Klein-Waardenburg syndrome) segregating with a heterozygous deletion in the paired box domain of PAX3: a simple variant or a true syndrome? Clin Genet. 2001; 60(4): 301–4. 11683776 10.1034/j.1399-0004.2001.600408.x

[63] Tassabehji M, Newton V, Liu X, The mutational spectrum in Waardenburg syndrome. Hum Mol Genet. 1995; (11): 2131–7. 8589691 10.1093/hmg/4.11.2131

[64] White D, Rabago-Smith M. Genotype-phenotype associations and human eye color. J Hum Genet. 2011; 56(1): 5–7. 20944644 10.1038/jhg.2010.126

[65] Edery P, Attié T, Amiel J, Mutation of the *endothelin-3* gene in the Waardenburg-Hirschsprung disease (Shah-Waardenburg syndrome). Nat Genet. 1996; 12(4): 442–4. 8630502 10.1038/ng0496-442

[66] Potterf S, Furumura M, Dunn K, Transcription factor hierarchy in Waardenburg syndrome: regulation of MITF expression by SOX10 and PAX3. Hum Genet. 2000;107(1): 1–6. 10982026 10.1007/s004390000328

[67] Nagy E, Maquat L. A rule for termination-codon position within intron-containing genes: when nonsense affects RNA abundance. Trends Biochem Sci. 1998; 23(6): 198–9. 9644970 10.1016/s0968-0004(98)01208-0

[68] Bondurand N, Dastot-Le Moal F, Stanchina L, Deletions at the *SOX10* Gene Locus Cause Waardenburg Syndrome Types 2 and 4. Amer J Hum Genet. 2007; 81(6): 1169–85. 17999358 10.1086/522090 PMC2276340

[69] Toma K, Rebbapragada I, Durand S, Identification of elements in human long 3’ UTRs that inhibit nonsense-mediated decay. RNA. 2015; 21(5): 887–97. 25805855 10.1261/rna.048637.114 PMC4408796

